# Christmas in the Hospitals

**Published:** 1916-12-30

**Authors:** 


### CHRISTMAS IN THE HOSPITALS.

### St. Thomas's Hospital.

CHRISTMAS at St. Thomas's was marked as usual at this great hospital, where the civil and military work go on side by side most harmoniously, by a happy spirit of quiet enjoyment, showing a full realisation of the strenuous times in which we live. Christmas cheer was abundant, and for the nonce the patients put aside their sufferings while the nursing staff and officers of the hospital sacrificed themselves even more fully than usual to make their 915 sick and wounded forget their sufferings for the day. In this they were much aided by the convalescent soldiers and the many talented visitors who gladly gave up their own home enjoyment to bring a ray of sunshine and hope into the hospital wards. As one poor man remarked to our representative, "A day like this does us more good than a week of ordinary treatment."

The wards were cheerful and beautifully decorated with flowers, but with none of the wasteful decorations of cotton-wool, etc. (only too precious at the present time), which has been known at some hospitals in the past. Christmas Day itself opened with early services in the hospital chapel, which was crowded by nurses, civil and military patients, and staff. A visit to the hospital kitchen between 11 A.M. and 12 noon made one realise the enormous amount of extra work willingly and ably undertaken by the kitchen staff. Turkeys and beef were provided in abundance, the former the gifts of friends, the wives of the members of the staff being particularly prominent in this good work; plum-puddings (provided on the military side by the *Daily Telegraph* and *Daily News* Funds) and fruits completed the meal. Means to drink the King's health were specially provided in every military ward. Dinner being cleared away and the necessary rest taken, the remainder of the afternoon was given up in all the wards to the entertainments which had been provided. Each patient had his own two friends at his bedside, and most tastefully decorated and lavishly provided tea-tables were in evidence on all sides.

It would be invidious to mention any particular entertainment where all worked so hard, but the concert provided by Miss Lilian Braithwaite and her talented friends may be mentioned.

By 8 P.M. all was quiet once more, and the hospital had settled to its night rest.

On Boxing Day the matron, Miss Lloyd Still, was "at home" to the old Nightingales, and then the probationers, admirably trained by Miss Coode, sang carols in the various wards, thus concluding a most happy Christmas time.

### St. George's Hospital.

ONE often hears the expression "How sad to have to spend Christmas in a hospital!" A visit paid to St. George's Hospital on Christmas Day is more likely to elicit the expression "How nice to spend Christmas in a hospital!" If it were not for the fact that sickness or injury is the cause of the patients' presence in the wards, it is highly probable that many more would like to spend as happy a time. At St. George's (where there are about 340 patients) the wards this year have not been decorated quite so lavishly as in other years. Those occupied by sick and wounded soldiers are the only ones decorated in the usual manner; whilst those occupied by the civilian patients have been made as bright as possible with plants and flowers.

On Christmas Day a dinner of roast beef and plum-pudding was provided for those able to partake of it. This was followed in the afternoon by what is known as the "Christmas tea" for the patients. Tea, coffee, fruit, crackers, etc., were provided, and the lady visitors and other friends present distributed useful and acceptable gifts to every patient. These gifts, with the decorations, crackers, etc., are provided from a special fund which is generously contributed by residents in the neighbourhood of the hospital. Those responsible for the welfare of the patients are thus enabled to do all that is possible to make the season a time of good will.

### GIFTS FOR THE CHILDREN.

In former years a Christmas-tree, very kindly sent from Scotland by one of the governors, was provided for the children in the board-room of the hospital, and gifts were distributed from it by one of the medical officers disguised as Santa Claus. This year the tree was dispensed with, and it was arranged that the children should receive their gifts from the lady visitors and others during the "Christmas tea." Those children, more especially the poorer ones, who may be unfortunate (or fortunate) enough to have to attend in the out-patient department about Christmas time also receive gifts provided from the special fund before mentioned.

Entertainments were not forgotten, and on Christmas Day Mr. William Rolfe gave a special one for the soldiers, for whom numerous gifts were sent, notably, chocolates from Messrs. Cadburys, tobacco and cigarettes from "Smokes, Soldiers and Sailors," and matches from Messrs. Bryant and May.

One of the governors of the hospital also arranged for a feast of "surprise" mince-pies. Each of the soldiers received a mince-pie, and in four of them was a surprise in the shape of 5s., the lucky finders naturally being the envy of their fellows.

Needless to say, the whole of the staff—medical, nursing, and lay—did their utmost, as usual, to impart a Christmas spirit throughout the institution. This is aptly illustrated by the action of a nurse, who came to the conclusion that the patients in one of the wards would derive some pleasure if they had a gramophone. She therefore set to work, and within a week obtained sufficient money from her own personal friends to obtain an excellent instrument and records.

### MRS. MARRIOTT'S BEQUEST.

It may not be generally known to all that St. George's has a country branch—namely, Atkinson Morley's Convalescent Hospital, Wimbledon, where there are one hundred beds. These are occupied by patients in varying stages of convalescence, about forty-five of them being soldiers. Here also Christmas is a time of cheerfulness and good will, and, as many of the patients are able to be out of bed, a more festive appearance is possible, especially in the day-rooms.

An excellent entertainment was arranged and gifts provided for the patients from a fund entitled "Mrs. Marriott's Bequest." Mrs. Marriott was the wife of a former chaplain to the hospital, and evidently believed in Christmas being made as bright and cheerful as possible, for when she died she left a sum of money to the

### CHRISTMAS IN THE HOSPITALS— (*continued*).

hospital and directed that the income should be used for the purpose of providing a Christmas entertainment for the patients.

### St. Mary's Hospital.

Here the Christmas festivities began early in the morning of Christmas Day, when every patient in the hospital received a suitable and serviceable present. Services were held in many wards so as to enable every-one who wished to receive the Holy Communion. The service in the chapel was fully choral. For weeks past the nurses had been practising carols under the able directorship of Miss Mollison, the home sister, and the house men attended the practices in the chapel. A double quartette sang two verses from "Sweeter than songs of summer." There was a large congregation, mostly wounded soldiers, who joined in the singing with great effect. The chapel was tastefully decorated by Miss Meyhew, the assistant matron.

#### Carols in the Hall.

Carol singing in the Entrance Hall followed the service. This was heard all over the hospital, to the great joy of the patients confined to bed. It was a touching sight to see so many wounded soldiers singing the carols with all their heart and soul. The poor fellows were so maimed and yet able to sing as heartily as if they had never faced the horrors of war.

The patients' dinner was served at 12 A.M., and consisted of turkey and plum-pudding, followed by fruit served in dainty paper dishes. The soldiers especially did justice to the good fare, and all the men were allowed to smoke. Pianos were placed in every ward and were going pretty well all day, except for a little time in the afternoon, when everyone had a rest. Visitors arrived at 3 P.M., and each patient was allowed to have one visitor to tea at 4 P.M.

#### The Decorations.

The wards were beautifully decorated, red being the favourite colour. Zunz Ward (where Sir Almroth Wright's wounded soldiers are all undergoing special treatment) had flags of all kinds arranged by the soldiers in a most artistic manner.

Lilian Holland, brilliant in red, had three snow scenes —Scotch, Welsh, and Russian respectively—in honour of the sister and nurses of these nationalities working in the ward. The chaplain offered a prize for the best decorated ward dressed entirely by the soldiers, which was won by Boynton Ward.

The ward entertainments commenced at 5 P.M. A troupe of residents dressed in character received great applause, the patients thoroughly enjoying seeing their own doctors dressed up. Four of the lady dressers, dressed also in character, were among the party.

Time would fail to tell of all the visitors who came in to help with the entertainments. Their reward must have been ample in the knowledge that all the patients had a really happy time and thoroughly enjoyed themselves.

### Hampstead Military Hospital.

Christmas Day at the New End Section of Hampstead Military Hospital was a great success. Everyone enjoyed it, and the decorations were very varied. A great deal was done by the patients, who showed wonderful originality. The "Ward of the Two Seasons"—winter merging into spring—was beautiful. "The House of Lancaster" was another striking ward, decorated with red roses and old embroidery. "Japan" was draped with genuine embroidery from the East, and had a pond with banks and flowers. In another ward a patient showed wonderful talent, and had painted screens and excellent copies of some of the most popular war pictures from *Punch*. A very realistic snow-storm fell in another ward, and a burning Zeppelin, being attacked by aircraft in the glare of a searchlight, was much admired. The "Hall of Fame" was very attractive, with beautiful festoons around the walls, and the names of our greatest and best, in wreaths of silver and gilt.

Dinner was served in each ward at 12.30. At the same time the Mayor of Hampstead and other guests visited all wards with hampers of presents.

At 2.30 a concert was held in the hall, followed by tea and a Christmas-tree. Music in the wards ended a very happy day for the "Tommies" at Hampstead.

### South London Hospital for Women: Its First Christmas.

The first Christmas celebrated in the South London Hospital for Women will be memorable for the succession of festivities which were arranged by the energy and good will of the matron, Miss Pearce, and her colleagues. Owing to the war these were of a quiet nature. On the afternoon of December 21 the students of the Royal Free School of Medicine kindly came to sing carols in the large wards, and some of the patients from the smaller wards and from the private paying wards were brought in to hear them. The lights were lowered, and the effect was made more impressive by the students carrying lanterns. On Christmas Day there were Services at 6.15 A.M. and at 11 A.M. In the early morning, after 7, two nurses, dressed as Santa Claus and his jester, made the round of the wards and distributed appropriate gifts and cards to each patient. Later in the day a Christmas dinner consisting of turkeys (provided by some of the medical staff) and plum-pudding was served to all who were able to enjoy it. During the afternoon a concert was given by members of the staff and friends, and each patient was allowed to have a visitor to tea, which was much appreciated.

Two pianos were kindly lent to the hospital by the Clapham High School for Girls. The wards were most tastefully decorated, and the white walls showed up the delicate colours of the flowers and the shades for the electric lights.

In the Queen Mary's Ward the shades were of pale blue paper ornamented with butterflies, made by the patients themselves, and baskets of pink and white flowers and ferns were hung at intervals along the ward.

In the large surgical ward the decoration was Japanese; each light was covered with a paper lantern, and branches of cherry-blossom and chrysanthemum, real and of paper, carried out the scheme.

The smaller wards and the private paying wards (all occupied) looked very bright and pretty.

On Boxing Day the nursing staff had their dinner and party, and on Wednesday the domestic staff had theirs. The turkeys for these dinners were also most generously provided privately.

A visit to all the wards on Tuesday afternoon showed a very happy set of patients, grateful for an enjoyable Christmas and for all that had been done for them in this newly-opened hospital.

### CHRISTMAS IN THE HOSPITALS—(*continued.*)

### The Leicester Hospitals.

The third "War" Christmas in these institutions was kept with cheerfulness and merriment, for, appropriately enough, the wounded soldier alone possesses the ability to appreciate the true spirit of the Christmas festival in these disturbing times. Essentially a home festival, a time of reunion of the family life, the characteristics of Christmas have temporarily departed, and have been for the time being transferred to institutions, especially to those undertaking the treatment of wounded sailors and soldiers. The reasons are not far to seek; in the homes thoughts naturally turn to those loved ones who are upholding the nation's honour in the stern fight for the maintenance of the liberty of smaller nations and to those who have fallen in the field. There is scarcely a home that does not come under the influence of such memories as these, for death has taken a heavy toll of sons, brothers, relatives, and friends, added to which the inconveniences and trials of present-day life tend to obscure the inward spirit of Christmas.

It is, happily, safe to say that these sad reminders of the world-wide war did not find their way into the hospitals, for those who have faced the actual horrors of warfare seem able to discover the source of brightness and cheerfulness, and to appreciate the advent of the time when lasting victory shall herald the approach of a brighter day and a period of good will. Yes, it was good to be in hospital; its brightness, its cheerfulness, and its comradeship are sadly missing in the national life to-day. And if the hospitals were such happy centres, then those upon whom devolve the arrangements for the festivities, and all who help in the provision of the good things that make for enjoyment, share in the spirit of festival. Assuredly, therefore, the people of Leicester spent a happy Christmas in the knowledge of the splendid provision made for the wounded soldiers who are temporarily the guests of the town. Ample reminders are not wanting in business life, and in hospital life, too, of an unexampled dearth of labour, but this was not so in the military hospitals. Willing hands and glowing hearts made light of such minor trifles as loss of limbs and wounds, and not a hospital or a convalescent home that did not show ample evidence of a concentrated purpose to make Christmas real by the provision of decorative schemes, many of which were of real merit. "Willing hearts make light work," and, it should be added, lighter hearts.

Actually the festivities commenced on Saturday afternoon, when 500 men were entertained to a tea and entertainment at the Borough Working Men's Club and Institute, followed in the evening by concerts and entertainments in the hospitals. The finishing touches to the decorations and final preparations were also made. On Sunday, at the Base Hospital, Holy Communion was celebrated at 6.15 and 7.15, followed by Parade Service at 11.15 and Free Church Service at 5.15. For carol-singing the help of church choirs was brought into requisition, St. Nicholas and St. Leonard for the Royal Infirmary and St. Peter and St. James for the Base Hospital.

Christmas Day was a day of days. Church Services were arranged as on Sunday. Dinner was served in the various wards, and, generally speaking, consisted of roast beef, *Daily News* and *Daily Telegraph* Christmas plum-pudding, custard, cheese, fruit, nuts, and crackers. The gracious message of His Majesty the King was received with much satisfaction and gave great pleasure.

#### Splendid Generosity.

One definite purpose stands out prominently in all these festivities—viz., the desire of the people of Leicester to provide the wounded with as much enjoyment and as much comfort as hearty good will and a sense of indebtedness for gallant service can render. All sections of the community combined in this one aim. School children set to work on decorative schemes and the provision of plum-puddings and other gifts. Workpeople contributed sums of money for the purchase of a hundred and one little delicacies which ordinarily military hospitals are precluded from providing. Ladies and gentlemen sent timely gifts of all kinds—game, cake, nuts, and presents. The War Hospitals Games Committee provided supplies of tobacco and cigarettes, which were supplemented by the generosity of the employees of Messrs. M. Wright and Sons, Ltd., of Quorn (who are engaged on Army work), who contributed a sufficient sum to provide each wounded soldier with 2 oz. of tobacco or twenty-five cigarettes as a special gift for Christmas Day. The West-End Association for the Entertainment of Wounded Soldiers presented a magic-lantern to the Base Hospital and six card-tables to the new recreation room for wounded soldiers at the Leicester Royal Infirmary. Messrs. Fielding and Johnson's employees undertook the provision of a billiard-table for the same object, and from a whist-drive and dance Mrs. Wood, Mrs. Smith, Mrs. Kellett, and Mrs. Taylor presented two spinal carriages, making, with one previously given, one each for the three military wards of this institution.

#### The Mayor and Mayoress's Constant Help.

Reference, too, must be made to the very real help of the Mayor and Mayoress, not only in connection with their Christmas gifts of iced cakes, etc., to all the institutions, but throughout the year in connection with the Mayoress's Equipment Fund. This Fund has provided over 3,000 articles for the hospitals, in addition to 1,600 articles made up from materials supplied by the Base Hospital. In addition it has undertaken all the mending of soldiers' garments, hospital linen, etc., averaging 800 pieces per week. The Fund has also undertaken the distribution of gifts to the various institutions, including thousands of eggs. Fourteen thousand mufflers, socks, etc., have been made up from materials supplied, and 1,600 garments have been sent to men engaged on mine trawlers, sweepers, etc., whilst 28,000 articles have been supplied by request of the War Department to Mesopotamia, Salonica, France, and various hospitals and depôts at home and abroad.

